# Abdominal Caesarian Sections

**Published:** 1915-05

**Authors:** 


**Abdominal Caesarian Sections** at Lying-In Hospital of the City of N. Y., Asa B. Davis, Bull. of the Lying-In Hospital, Feb. 1915. In about 80,000 deliveries, section has been performed 571 times, with a maternal mortality of 10.7%—13 of the 61 deaths being due to toxaemia or eclampsia, reducing the actual mortality of the operation to at most 8%. In 121 contrasted craniotomies, the death rate was 15.5%. Of the 577 children delivered 23 (4%) were still-born, 46 (8%) died. 35 cases were indicated by eclampsia and toxaemia of pregnancy, 37% maternal deaths, occurring. From these 35 women, 37 children were born, 4 still-born and 7 died, total infantile mortality 30%.

Placenta praevia was the main indication in 21 cases, 2 mothers died of sepsis (10%), 21 children were born, 3 still-born and 4 died, total infantile mortality 33 1/3%.

Accidental haemorrhage occurred in three deliveries, no maternal deaths, one child still-born, one premature and died shortly.

78 cases of repeated Caesarian section occurred, 60 of the second time, 15 for the third, 1 each for the fourth, fifth and sixth time. Rupture of the uterus in a labor subsequent to one at which Caesarian section had been performed, occurred 6 times, with a mortality of 3 mothers. Of the 6 children, four were free in the abdominal cavity and already dead, the others were extracted and lived.

441 cases or 79% were due to contracted pelvis or some deformity of the spine, resulting in faulty presentation. Malecostion, considered a common indication by Playfair, was encountered only once. There were 512 vertex presentations, 2 brow, 2 face, impacted, 2 transverse, 2 of prolapsed cord and 24 in which the presentation was not noted. 214 cases were operated at the first labor, 128 at the second, 91 at the third, 45 at the fourth, 47 at the fifth, 13 at the sixth, 14 at the seventh, 13 at the eighth, 4 at the ninth, 3 at the tenth, 2 at the twelfth and one each at the thirteenth and fourteenth.

